# Digital endpoints in clinical trials of Alzheimer’s disease and other neurodegenerative diseases: challenges and opportunities

**DOI:** 10.3389/fneur.2023.1210974

**Published:** 2023-06-15

**Authors:** Anna-Katharine Brem, Sajini Kuruppu, Casper de Boer, Marijn Muurling, Ana Diaz-Ponce, Dianne Gove, Jelena Curcic, Andrea Pilotto, Wan-Fai Ng, Nicholas Cummins, Kristina Malzbender, Vera J. M. Nies, Gul Erdemli, Johanna Graeber, Vaibhav A. Narayan, Lynn Rochester, Walter Maetzler, Dag Aarsland

**Affiliations:** ^1^Department of Old Age Psychiatry, King’s College London, Institute of Psychiatry, Psychology and Neuroscience, London, United Kingdom; ^2^University Hospital of Old Age Psychiatry, University of Bern, Bern, Switzerland; ^3^Alzheimer Center Amsterdam, Neurology, Vrije Universiteit Amsterdam, Amsterdam UMC Location VUmc, Amsterdam, Netherlands; ^4^Amsterdam Neuroscience, Neurodegeneration, Amsterdam, Netherlands; ^5^Alzheimer Europe, Luxembourg, Luxembourg; ^6^Novartis Institutes for Biomedical Research (NIBR), Basel, Switzerland; ^7^Neurology Unit, Department of Clinical and Experimental Sciences, University of Brescia, Brescia, Italy; ^8^Laboratory of Digital Neurology and Biosensors, University of Brescia, Brescia, Italy; ^9^Neurology Unit, Department of Continuity of Care and Frailty, ASST Spedali Civili Brescia Hospital, Brescia, Italy; ^10^Translational and Clinical Research Institute, Newcastle University, Newcastle upon Tyne, United Kingdom; ^11^NIHR Newcastle Biomedical Research Centre and Clinical Research Facility, Newcastle upon Tyne Hospitals NHS Foundation Trust, Newcastle upon Tyne, United Kingdom; ^12^Department of Biostats and Health Informatics, King’s College London, Institute of Psychiatry, Psychology and Neuroscience, London, United Kingdom; ^13^Gates Ventures, Kirkland, WA, United States; ^14^Foundation Lygature, Utrecht, Netherlands; ^15^Novartis Pharmaceuticals Corporations, Cambridge, MA, United States; ^16^Institute of General Practice, University Medical Center Schleswig-Holstein, Kiel University, Kiel, Germany; ^17^Davos Alzheimer’s Collaborative, Geneva, Switzerland; ^18^Faculty of Medical Sciences, Translational and Clinical Research Institute, Newcastle University, Newcastle upon Tyne, United Kingdom; ^19^National Institute for Health and Care Research (NIHR) Newcastle Biomedical Research Centre (BRC), Newcastle University and The Newcastle upon Tyne Hospitals NHS Foundation Trust, Newcastle upon Tyne, United Kingdom; ^20^Department of Neurology, University Hospital Schleswig-Holstein and Kiel University, Kiel, Germany; ^21^Centre for Age-Related Medicine, Stavanger University Hospital, Stavanger, Norway

**Keywords:** Alzheimer’s disease, Parkinson’s disease, Huntington’s disease, neurodegenerative diseases, dementia, digital biomarker, remote measurement technologies, digital health technologies

## Abstract

Alzheimer’s disease (AD) and other neurodegenerative diseases such as Parkinson’s disease (PD) and Huntington’s disease (HD) are associated with progressive cognitive, motor, affective and consequently functional decline considerably affecting Activities of Daily Living (ADL) and quality of life. Standard assessments, such as questionnaires and interviews, cognitive testing, and mobility assessments, lack sensitivity, especially in early stages of neurodegenerative diseases and in the disease progression, and have therefore a limited utility as outcome measurements in clinical trials. Major advances in the last decade in digital technologies have opened a window of opportunity to introduce digital endpoints into clinical trials that can reform the assessment and tracking of neurodegenerative symptoms. The Innovative Health Initiative (IMI)-funded projects RADAR-AD (*Remote assessment of disease and relapse—Alzheimer’s disease*), IDEA-FAST (*Identifying digital endpoints to assess fatigue, sleep and ADL in neurodegenerative disorders and immune-mediated inflammatory diseases*) and Mobilise-D (*Connecting digital mobility assessment to clinical outcomes for regulatory and clinical endorsement*) aim to identify digital endpoints relevant for neurodegenerative diseases that provide reliable, objective, and sensitive evaluation of disability and health-related quality of life. In this article, we will draw from the findings and experiences of the different IMI projects in discussing (1) the value of remote technologies to assess neurodegenerative diseases; (2) feasibility, acceptability and usability of digital assessments; (3) challenges related to the use of digital tools; (4) public involvement and the implementation of patient advisory boards; (5) regulatory learnings; and (6) the significance of inter-project exchange and data- and algorithm-sharing.

## 1. Introduction

Digital endpoints in clinical trials are being investigated increasingly in large-scale international projects. The rapid advancement of technological developments allows entirely new approaches to assessing activities of daily living (ADL), sleep and fatigue, motor, cognitive, social, neuropsychiatric, and autonomous body functions with potential for both trials and clinical practice. The appeal lies in the objective, immediate and continuous measurement in both clinical and home settings, the reduction of visits to research or clinic facilities, the accessibility for under-served populations, the potential for better stratification and more personalised therapies, and the possibility to support otherwise time-intense clinical decisions with Artificial Intelligence (AI). This is of specific importance for Alzheimer’s disease (AD), but also other neurodegenerative diseases, such as Parkinson’s disease (PD) and Huntington’s disease (HD), with a predominantly slow progression over years as well as cognitive impairment and fluctuations, which reduce the validity of data from self-rated or one-time assessments.

Functional decline is a significant indicator of progression of neurodegenerative diseases. A range of questionnaires have been developed to assess ADL (1). However, many of these instruments lack sensitivity to change in early stages of a disease and therefore have a limited utility as outcome measures in clinical trials (2, 3). This is of specific importance considering recent developments in disease-modifying drugs for the treatment of AD, such as aducanumab and lecanemab (4) that are targeting early cognitive impairment and emphasise the need for highly sensitive methods. Similar restrictions apply to standard mobility and neuropsychological testing and the query of social skills, sleep, fatigue, neuropsychiatric symptoms, and autonomous body functions with self- and informant-rating questionnaires. Standard assessments are intermittent, costly, and partly rely on subjective information, which is especially problematic in later stages of a neurodegenerative disease. The common goal of the Innovative Health Initiative (IMI)-funded projects RADAR-AD, IDEA-FAST and Mobilise-D is to define digital endpoints relevant for neurodegenerative diseases that provide reliable, objective, and sensitive evaluation of disability, ADL, and health-related quality of life.

**RADAR-AD** (EC Grant No.806999; www.radar-ad.org) aims to identify and validate remote monitoring technologies (RMTs) to assess functional impairment in all stages of Alzheimer’s disease. The study includes wearables and smartphone apps in the main study (*n* = 232) and passive at-home sensors in a sub-study (*n* = 45). The RMTs measure a wide range of cognitive and functional domains, including spatial navigation, activity, sleep, speech, driving behaviour, and gait (5).

**IDEA-FAST** (EC Grant No. 853981; www.idea-fast.eu) aims to identify digital parameters in patients with PD and HD, and immune-mediated disorders, which are related to fatigue, sleepiness, and sleep quality. A pilot study (6, 7) has informed the design of a larger clinical observational study using different devices concurrently to capture data on ADL-related activities, sleep, physiological and cognitive/psychological variables. In the latter study, up to 2000 participants (PD *n* = 500; HD *n* = 200) will be recruited at up to 24 sites across Europe.

**Mobilise-D** (EC Grant No. 820820; www.mobilise-d.eu) (8) aims to validate a suite of digital mobility outcomes to directly monitor mobility performance continuously over a 7 day duration using a single wearable device in PD (*n* = 600) and other diseases associated with mobility impairment (chronic obstructive pulmonary disease, multiple sclerosis, proximal femoral fracture) (9, 10).

**Figure 1:**
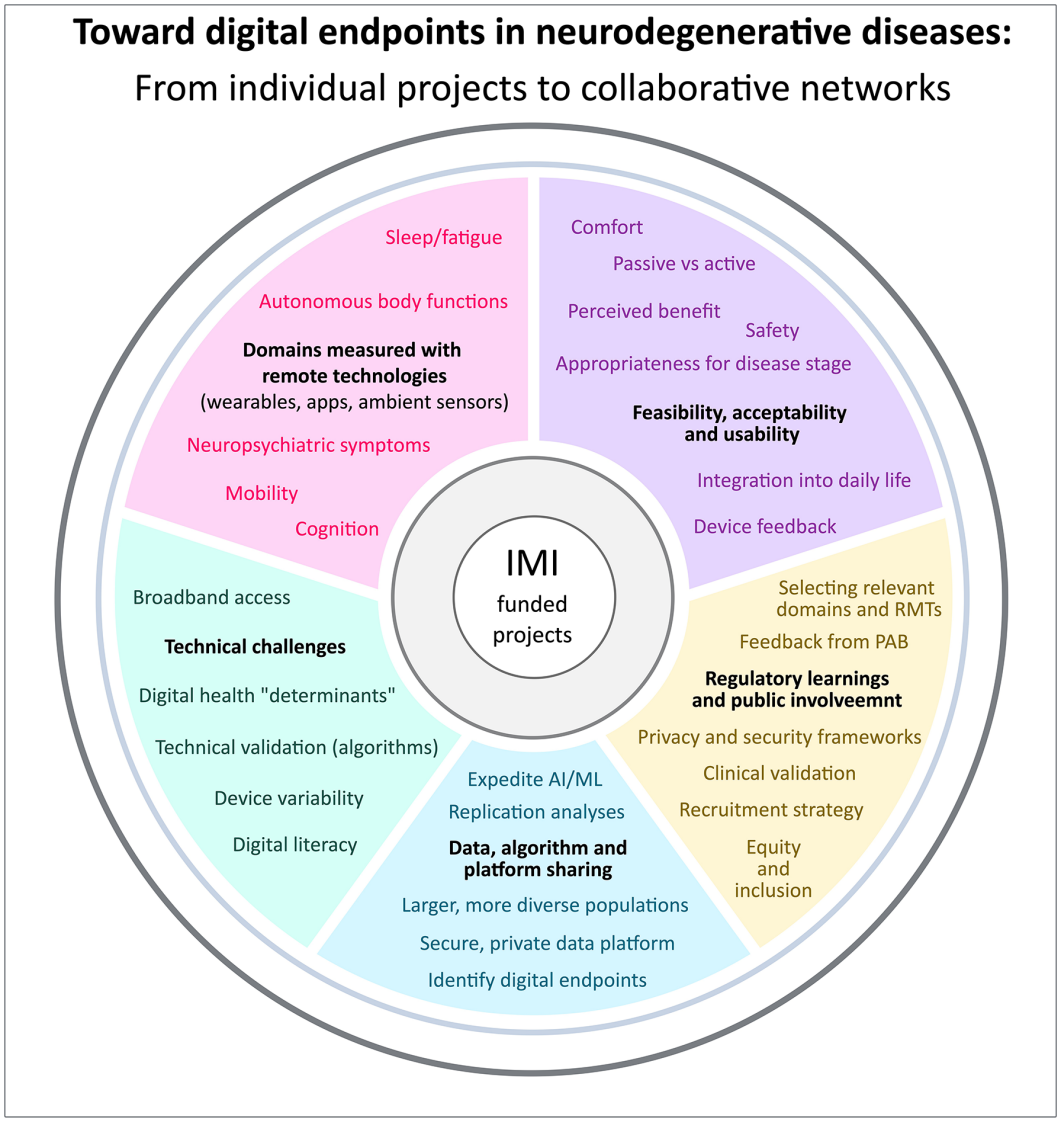
Findings and experiences of RADAR-AD, IDEA-FAST, and Mobilise-D in (1) remote technologies to assess neurodegenerative diseases, (2) feasibility, acceptability and usability of digital assessments, (3) challenges related to the use of digital tools, (4) regulatory learnings and public involvement, and (5) data, algorithm and platform sharing.

In this article, we will draw from the findings and experiences of these different IMI projects in discussing (1) the value of remote technologies to assess neurodegenerative diseases; (2) feasibility, acceptability and usability of digital assessments; (3) challenges related to the use of digital tools; (4) public involvement and the implementation of patient advisory boards to guide clinical trials in terms of protocol design, ethical issues, and selection and applicability of digital tools; (5) regulatory learnings; and (6) the significance of inter-project exchange and data- and algorithm-sharing (Figure 1).

## 2. The value of remote technologies to assess neurodegenerative diseases

Technological advances in the last decade opened a window of opportunity to introduce digital endpoints into clinical trials. RMTs could provide a useful, objective way to measure decline by collecting data that correspond to various functional domains that are clinically relevant. They assess functional ability either passively (i.e., not requiring any interaction with the device, such as is the case with gait measures) or interactively (i.e., requiring an active interaction with the device such as when assessing functional abilities involving cognition). The benefit of RMTs as compared to standard assessments, is that they are objective and can collect data in the real world continuously. They are ideally placed to potentially measure subtle functional changes that are prevalent among individuals in the early, preclinical stages of neurodegenerative diseases, where current methods of cognitive assessments lack the necessary sensitivity (11) and to continuously track changes during the course of a disease. The RMTs used in the different consortia are listed in Table 1.

**Table 1:** Domains assessed in the three IMI-funded consortia RADAR-AD, IDEA-FAST, and Mobilise-D.

	Cohorts	Trial design	Domains assessed	Domains assessed digitally
RADAR-AD	HC *n* = 70 PreAD *n* = 38 ProAD, *n* = 65 MildAD *n* = 56	8 W observation period	Activities of daily living Cognitive functions Sleep quality and fatigue Life habits Mobility Social functioning Smartphone proficiency Quality of life Neuropsychiatric symptoms including depression Medical history and medication Physical examination	Activities of daily living (apps and wearables) Cognition (apps) Sleep and circadian rhythm (wearables, sleep EEG) Mood and fatigue (app) Mobility SS assessment (IMU) Mobility US assessment (wearables) Social (app) Driving (data logger)
Mobilise-D	PD *n* = 600 MS *n* = 600 COPD *n* = 600 PFF *n* = 600	1 W observation period every 6 M (5 times in total per participant)	Risk of falls Cognitive functions BIA Fatigue Disability Pain Frailty Severity of specific conditions	Mobility SS assessment (6MWT, TUG) Mobility US assessment (IMU)
IDEA-FAST	HC *n* = 200 PD *n* = 500 IBD *n* = 500 RA *n* = 200 SLE *n* = 200 PSS *n* = 200	1 W observation period every 6 W (4 times in total per participant)	Sleep quality Fatigue (mental vs. physical) Cognitive screening Disability Pain Severity of specific conditions	Mobility US assessment Sleep (bed sensors, sleep EEG) ECG and autonomic function Fatigue (app) Cognition (app) Social (app)

AD, Alzheimer’s disease; BIA, body impedance analysis; COPD, Chronic Obstructive Pulmonary Disease; ECG, electrocardiography; EEG, electroencephalography; HC, healthy controls; IBD, Inflammatory bowel disease; IMU, inertial measurement unit; M, months; MildAD, mild-to-moderate AD (dementia, Aβ-positive); MS, multiple Sclerosis; PD, Parkinson’s disease; PFF, Proximal Femur Fracture; preAD, preclinical AD (cognitively normal, Aβ-positive); proAD, prodromal AD (mild cognitive impairment, Aβ-positive); PSS, Primary Sjogren’s syndrome; RA, Rheumatoid Arthritis; SLE, Systemic Lupus Erythematosus; SS; supervised setting; TUG, timed up and go test; US unsupervised setting; W, week(s); 6MWT, 6 min walking test.

In the three consortia, different functional domains were measured. Mobility, for example, was evaluated in various ways within the IMI projects. Mobilise-D applied both supervised (in the presence of study staff) and unsupervised testing using a standardised protocol. In addition to that, home mobility was evaluated using an inertial measurement unit (IMU) for 7 days at different time points. In RADAR-AD, mobility was evaluated using a supervised standardised protocol as well, and home mobility using a wrist-worn IMU for 8 consecutive weeks. In both RADAR-AD and IDEA-FAST, heart rate was measured using a wearable. Another functional domain assessed was sleep. IDEA-FAST and RADAR-AD both made use of an app to actively collect data on sleep, asking the participants daily about their fatigue, sleep pattern and quality. Moreover, sleep was measured passively: RADAR-AD made use of a portable EEG device, which a subset of participants wore every night for a month, while IDEA-FAST used a bed sensor with a force-sensitive piezo-electric film, placed under the mattress. Cognition has been addressed in a supervised standardised way in all consortia. Cognitive data was evaluated remotely using several smartphone apps in RADAR-AD and a web-based application of CANTAB in IDEA-FAST and both consortia collected passive information on smartphone use, including keyboard metrics and GPS location tracking.

Future clinical trials will profit from these recent technological developments, which promise improved sensitivity and specificity of endpoint measures, better external validity, and the need of fewer visits to research or clinical facilities and smaller sample sizes due to more detailed datasets per participant.

## 3. Challenges related to the use of digital tools

The use of RMTs can present challenges with respect to a range of aspects including the validity of measurements, related to sensitivity and specificity (e.g., differentiating sensor information in multi-person households), data quality, e.g., choosing the right time granularity (12), data missingness, which is often due to technical and software issues (13), and subsequent analysis. The use of Artificial Intelligence (AI) to combine and analyse RMT signals brings a multitude of challenges itself (14), including privacy and security concerns (15), gaining informed consent (16), and ethical challenges. These can be addressed by creating regulatory frameworks and promoting public-private partnerships (17). Ensuring equity and inclusion when deploying digital tools is another important challenge. Connectivity and broadband access, device variability/obsolescence and digital literacy are “digital determinants of health” that impact equitable access to digital healthcare and the outcomes from and experience with digital tools (18). To date, 37% of the world’s population has never used the internet. In the European Union, the percentage of older people (aged 65–74) using the internet varies greatly from 25% in Bulgaria to 94% in Denmark and we face a growing age gap in smartphone ownership in emerging economies around the globe (19). Even if a smartphone or PC is available in a household, access might still be restricted due to financial or technical reasons (20). Digital health studies have developed approaches such as “bring-your-own-device studies” (21), providing funding for internet connectivity, or using sensors that are not (continuously) connected to the internet to help address these challenges. Collocation and sharing of best practices across projects will help address these challenges.

## 4. Feasibility, acceptability, and usability of digital assessments

It becomes increasingly important to consider the feasibility, acceptance, usability, and ecological validity of digital endpoints in real-world settings. Few studies report on these factors and ageing populations are not well represented in RMT research (22), but are explored in RADAR-AD, IDEA-FAST and Mobilise-D in collaboration with patients and carers. In studies involving wearables and smartphone apps, acceptance to use devices and adherence to protocol are in general positive when they are reported (23, 24). For example, the comfort and acceptability of a wearable sensor to monitor mobility in the Mobilise-D study was very high (23). However, many studies to date lack information on acceptability, adherence and usability (24). Overall, passive devices/apps requiring little or no interaction with a device show higher feasibility, acceptability and usability than interactive devices and are the most researched to date (25). Research in PD reports that the successful implementation of digital technologies is primarily driven by familiarity with the technology and ease of use, costs, motor symptoms hampering the use, experiencing beneficial effects, and feeling safe whilst using the technology (26). In AD, acceptance and adherence can be facilitated by familiarising participants with the devices and providing personal support, lowering technical demands, co-designing solutions and involving relevant stakeholders, introducing participants to the devices at the earliest stages of the disease, and increasing the perception of effectiveness and safety. Barriers mainly include technology anxiety, system failures, and lack of access (27, 28). However, if these factors are addressed, adherence is generally high (85.7%) in older adults (29).

Some of these barriers became apparent in RADAR-AD. For example, engaging with RMTs led to some participants feeling discouraged, as they acted as a reminder for their declining cognition. Cognitive impairment also led to missing data, e.g., participants removed their wearables before going to bed, meaning sleep hygiene could not be tracked. Study partners are essential when it comes to reducing or overcoming (cognitive) barriers—they help with charging/handling RMTs, provide emotional support, and remind participants to keep wearing/using RMTs. Overall, study partners are vital in the adherence and usability of digital tools in neurodegenerative diseases (Muurling et al., submitted)^1^. In RADAR-AD and IDEA-FAST, participants reported adjustments to daily routines; specifically, acclimating to wearing two wrist-worn wearable devices, using their phone more, and adjusting personal schedules to complete their daily app-based tasks on time. Ergonomic challenges were reported due to the physical design of watches (i.e., watch straps not fitting well or feeling limited in their movements). Similar findings have been collated within multiple systematic reviews on digital tool use in older adults (28, 30, 31). Participants reported individual preferences for the display of the wearable screen (e.g., matching the clock face of their usual watch) and for device feedback (e.g., cognition and activity tracking), which facilitated integration into daily routines. Lack of, or inaccurate device feedback, small screens and small fonts also contributed towards the challenges faced by participants. In the IDEA-FAST pilot study, participants moreover mentioned skin irritations due to adhesive patches, constant worry about the device and insecurities regarding its proper functioning. Also, participants reported being less willing to wear devices that were very visible, complicated to use, or that had to be manipulated at impractical times, e.g., right before sleeping. The roadmap towards translating RMT use from research to clinical practice has to continue to evolve, together with patient and stakeholder involvement, as the benefits and challenges are evaluated (32).

## 5. Public involvement and the implementation of patient advisory boards

Public Involvement (PI) is about involving people affected by the condition in all aspects of the research process as partners rather than as research participants (33, 34). PI not only provides the patients’ perspective on what research is important and which unmet needs should be addressed, but it is also about understanding and anticipating what aspects of the research may be difficult to manage by the participants, may raise concerns, and how these issues could be addressed. It also involves reflecting about future issues, challenges, and benefits of the project, if and when the results are eventually implemented in the real world. Involving people from minority ethnic groups and other under-served populations is crucial but still remains a challenge (33).

All three consortia involved patients and, in the case of RADAR-AD, also carers in special advisory boards. They provided strategic input to various aspects of consortium activities throughout the projects, including: study protocols and participant-facing documents; digital health technology in general and digital assessments and outcomes in particular; feasibility, usability and acceptability of digital outcome assessment and how it can contribute to improved care; consultation around health technology assessment and regulatory acceptance of digital outcomes; ethical considerations, recruitment and retention strategies; and involvement in promotion activities about the impact and benefits of results. RADAR-AD and IDEA-FAST also collaborated with patient organisations and in IDEA-FAST, two additional groups consisting of patients, consortium members and representatives from patient organisations were formed to develop and review the project activities and to support the design of the two clinical studies.

## 6. Regulatory learnings

If digital endpoints are to be used in clinical trials aimed to achieve a market authorisation for medicinal products, it is of paramount importance that the endpoints are accepted by the regulatory authorities. In recent years, the use of RMT-based assessments has increased dramatically (35). However, the number of digital endpoint measures that are qualified is still limited (36) and there are no approved primary or secondary digital endpoints for use in clinical trials in AD or PD yet (35, 37). In RADAR-AD, a regulatory strategy was developed early on, including an extensive evaluation of all qualification opinions and advices and scientific advices of the EMA to gain insight in the types of tools that are intended to be used in clinical trials for supporting/submitting applications for obtaining market authorization (registration trials) (36). The EMA recommendations evolved mainly around the relevance, precision, and accuracy of novel endpoints; validation with current gold standards and clinically meaningful legacy endpoints, including those that matter most to patients (“daily-relevant data”); sensitivity and specificity; good compliance and acceptability; and guarantee of optimal data security and privacy. The RADAR-AD consortium had an initial meeting with the Innovation Task Force in 2020 and is currently in the process of having a Qualification Advice discussion with EMA. The Mobilise-D consortium had two consecutive EMA qualification advices in 2020 (38, 39) and a letter of support was published on the EMA website (40, 41) following each qualification advice. Mobilise-D has furthermore interacted with the Food and Drug Administration (FDA). The IDEA-FAST consortium had two meetings with the EMA between 2020 and 2022. The first meeting with the Innovation Task Force was to discuss the general concepts of developing digital endpoints for fatigue and sleep. The second meeting was to discuss the study design and data analytic plan of a clinical study to identify these digital endpoints which was given general support by the Scientific Advice Working Party.

It is highly recommended for similar consortia to develop a regulatory strategy early on, to ensure that what is being developed will also be accepted in drug trials. It is important to plan for multiple Health Authority meetings utilising Innovation Task Force and EMA Qualification advice meetings as well as meetings with other major Health Authorities, as appropriate. Early advice on study design prior to protocol finalisation/study initiation would be highly desirable. Further development of clear guidance for the use of digital technologies in registration trials could remove some of the regulatory hurdles that currently complicate the development and use of novel improved endpoints (42).

## 7. The significance of inter-project exchange and data- and algorithm-sharing

To extend and generalise individual project findings and foster deeper understanding of digital outcomes across neurodegenerative diseases, inter-project exchange and data sharing has gained significance. The full value of data collected in large research programmes can only be realised by enabling a wider set of analytics than is possible through individual consortia. This need is only heightened by the current rapidly expanding popularity in AI and Machine Learning research which relies on large datasets. Sharing resources allows for more rapid research to be undertaken, leading to greater efficacy in terms of advancing state-of-the-art than could be otherwise be achieved working on the data in isolation. For example, the sharing of speech data through DementiaBank (43) has enabled a wide range of different machine learning approaches to be compared and assessed on a common database (44). In such a rapidly growing area of research it is also important to conduct replication analysis and robust generalised testing of proposed digital phenotypes. Sharing and open sourcing algorithms enables these vitally important verification steps.

The sharing of data requires careful considerations to preserve the privacy of participants in a manner that not only meets ethical and statutory requirements, but also meets participants’ expectations regarding distribution of their data. Entire IMI-projects have developed around this topic. For example, the European Platform for Neurodegenerative Diseases (EPND, www.epnd.org) aims to accelerate the discovery of diagnostics and treatments for neurodegenerative diseases by removing barriers to data and sample sharing (45). This includes sharing of digital data, by building a robust and secure data sharing infrastructure and funding a case study of prospective digital (bio)marker data collection. EPND aims to build connections to existing data platforms and facilitate the discoverability of resources; provide secure, private cloud-based workspaces where researchers can perform and save analyses; collaborate with other permissioned users; and develop ethical, legal, and regulatory principles guiding platform design and discovery and sharing of data.

The access to and reuse of research data generated by Horizon 2020 projects is available through the Open Research Data Pilot (ORD Pilot), which is in line with the FAIR (Findable, Accessible, Interoperable, Reusable) principles^2^ and ensures open access to publications and research data (curated and raw data) including access to, e.g., specialised software or software code, algorithms, and analysis protocols. This allows to build on previous research findings, foster collaboration, promote innovation, and improve transparency in research (46). New projects can be greatly strengthened by reusing infrastructure, such as RADAR-base, and sharing algorithms between consortia that use similar RMTs, such as RADAR-CNS, in the case of RADAR-AD.

We argue that sustainability should be plannable and funded beyond the duration of a project, ideally via IMI-funded platforms, to guarantee a lasting impact and allow following projects to profit from the large data volumes produced by RMTs, previous experiences, including cross-learning about device selection and barriers/facilitators of using digital health technology, especially for studies that are targeting similar demographics and conditions.

## 8. Conclusion

Technological advances and collaboration between IMI-funded and other consortia bring new opportunities to develop and introduce digital endpoints into clinical trials that can revolutionise the assessment and tracking of neurodegenerative symptoms. The digitalization of endpoints allows for objective, immediate and continuous measurement in both clinical and home settings, the reduction of visits to research or clinic facilities, greater accessibility for under-served populations, better stratification and more personalised interventions, and AI-supported clinical decisions.

## RADAR-AD consortium

Dag Aarsland, Halil Agin, Vasilis Alepopoulos, Alankar Atreya, Sudipta Bhattacharya, Virginie Biou, Joris Borgdorff, Anna-Katharine Brem, Neva Coello, Pauline Conde, Nick Cummins, Jelena Curcic, Casper de Boer, Yoanna de Geus, Paul de Vries, Ana Diaz, Richard Dobson, Aidan Doherty, Andre Durudas, Gul Erdemli, Amos Folarin, Suzanne Foy, Holger Froehlich, Jean Georges, Dianne Gove, Margarita Grammatikopoulou, Kristin Hannesdottir, Robbert Harms, Mohammad Hattab, Keyvan Hedayati, Chris Hinds, Adam Huffman, Dzmitry Kaliukhovich, Irene Kanter-Schlifke, Ivan Koychev, Rouba Kozak, Julia Kurps, Sajini Kuruppu, Claire Lancaster, Robert Latzman, Ioulietta Lazarou, Manuel Lentzen, Federica Lucivero, Florencia Lulita, Nivethika Mahasivam, Nikolay Manyakov, Emilio Merlo Pich, Peyman Mohtashami, Marijn Muurling, Vaibhav Narayan, Vera Nies, Spiros Nikolopoulos, Andrew Owens, Marjon Pasmooij, Dorota Religa, Gaetano Scebba, Emilia Schwertner, Rohini Sen, Niraj Shanbhag, Laura Smith, Meemansa Sood, Thanos Stavropoulos, Pieter Stolk, Ioannis Tarnanas, Srinivasan Vairavan, Nick van Damme, Natasja van Velthogen, Herman Verheij, Pieter Jelle Visser, Bert Wagner, Gayle Wittenberg, and Yuhao Wu.

## Mobilise-D consortium

Full membership of the Mobilise-D consortium is available on the website http://mobilise-d.eu/wp-content/uploads/2023/06/v9-logos_06.17.2022_Mobilise-D-consortium-members-names.pdf.

## IDEA-FAST consortium

Wan-Fai Ng, Christopher Bull, John Isaacs, Chris Lamb, Alison Yarnall, Lynn Rochester, Silvia Del Din, Chloe Hinchliffe, David Halliday, Ashur Rafiev, Bing Zhai, Dan Jackson, Peter Gallagher, Victoria MacRa, Leigh Denley, Ellen Silva, Philip Brown, Helen Gallon, Sean Scott, Phillip McGrouther, Dean Miller, Darren Storey, Lee Briton, Walter Maetzler, Kirsten Emmert, Robert Goeder, Jennifer Kudelka, Corina Maetzler, Hanna Kaduszkiewicz, Tanja Lange, Marie Bornhorst, Hanna Grasshoff, Stefan Schreiber, Sophia Hinz, Friso Muijsers, Kristina Brandt, Tina Hagen-Hurley, Robbin Romijnders, Clint Hansen, Linda Pialek, Kirstin Hansen, Johanna Graeber, Susanna Nikolaus, Florian Schrinner, Pia Görrissen, Paula Cullen, Maren Williams, Andrea Pilotto, Alessandro Padovani, Sabrina Denardi, Giulio Bonzi, Marcello Catania, Valentina Chirico, Fiorenza Cavagnini, Christen Janneke van de Woude, Nynke Borren, Monique Devillers, Nicole Larmonie, Denise Schenk, Hans van Leeuwen, Jorina van der Salm, Iain McInnes, Neil Basu, Joe Galloway, Norelee Kennedy, Alexander Fraser, Hayley Connolly, Sadhbh Ni Mhidigh, Imelda Doolan, Cathal Linnane, Jacques Demotes-Mainard, Linda Stöhr, Neshat Chareh, Hanna Schrinner-Fenske, Martina Esdaile, Alicja Szofer-Araya, Costantino Pitzalis, Michele Bombardieri, Louise Warren, Myles Lewis, Paul Giuliani, Sharon Palmer, Vicky Byers, Yi-Ke Guo, Kai Sun, Danilo Mandic, Mary Morrell, Siyao Wang, Florian Guitton, Yifeng Mao, Ailsa Hart, Shaun Power, Guanyu Tao, Benjamin Vandendriessche, Arno Bossaert, Rebeca Munoz, Hans De Clercq, Pierrick Arnal, Bertrand Fatus, Evgeniia Kurash, Maya Dorsey, Miles Parkes, Sree Subramanian, Louise Stockley, Rona Smith, Renata Schaeffer, Jérôme Kalifa, Jonathan Chauvin, Clémence Pinaud, Adrien Bennetot, Alexandra Belfiore, Laura Carrasco Marin, Mayca Marín, Jennifer Jimenez, Miriam Grande, Susana Donate, Evert-Ben van Veen, Daniel Groos, Martin Boeckhout, Beatrice van der Velden, Olenka van Ardenne, Denis Groot, Nebo Savic, Irene Schluende, Franziska Klepka, Simon Beniston, Veli Stroetmann, Rainer Thiel, Daniel Schmidtmann, Karin Breuer, Jessica Paul, Shahan Tariq, Alexandra Prodan, Griselda Marku, Tiago Guerreiro, André Rodrigues, Diogo Branco, Livia Moreira, Carla Marques, Hélia Rodrigues, Fabio Roli, Davide Ariu, Stefania Casula, Battista Biggio, Luca Piras, Luca Didaci, Matteo Mauri, Joaquim Ferreira, Leonor Correia Guedes, Inês Dias, Ana Teresa, Joana Costa, Mariana Matos, Teemu Ahmaniemi, Luc Cluitmans, Jani Mäntyjärvi, Juha Kortelainen, Rajdeep Nath, Emmi Antikainen, Kinga Koski, Francesca Cormack, Michele Veldsman, Laura Keylock, James Dobson, Janet Griffiths, Nick Taptiklis, Julian Fierrez, Aythami Morales, Ruben Tolosana, Alejandro Pena, Luis Gomez, Rafael Oliveros, Ruben Vera-Rodriguez, DaQing Zhang, Xujun Ma, Mossaab Hariz, Pei Wang, Djamal Zeghlache, Jordi Evers, Laura Siepman, Martijn Niessen, Ralf Reilmann, Robin Schubert, Atbin Djamshidian-Tehrani, Grzegorz Witkowski, Halina Sienkiewicz-Jarosz, Malgorzata Dusza-Rowinska, Klaus Seppi, Katarina Schwarzova, Corinne Horlings, Samuel Labrecque, Anita Malik, Wolfram Rieneck, Maria B. Lauvsnes, Roald Omdal, Katrine Norheim, Svein Skeie, Anne Hjelle, Hilde Norvik, Dave Wenn, Mike Jackson, Luisa Avedano, Bella Haaf, Tatiana Negurita, Susanne de Bot, Carola Buitelaar, Kasper van der Zwaan, Laura Kuijper, Adrie van Weeghel, Ian Bruce, John McBeth, Liz Fay, Joanna Jozefiak, James Prior, Denise Faulkner, Ioannis Pandis, Nikolay Manyakov, Stefan Avey, Meenakshi Chatterjee, Kenneth Mosca, Cesar Calderon, Rana Rehman, Melissa Mendez-Nguyen, Lori Warring, Marc Walton, Bethany Paxson, Diana Koletzki, Shyla Jagannatha, Zhi (Carrie) Li, Drew Elias, Kai Langel, Dario Masullo, Matthew Roche, Victoria Zolfaghari, Sarah Weingast, Maurizio Facheris, Tony Bannon, Matt Czech, Jie Shen, Shiv Patel, Michelle Crouthamel, Josh Cosman, Sean Turner, Magnus Jörntén-Karlsson, Tim Ruckh, Folke Folkvaljon, Jörgen Jensen, Neil Newman, Susan Forda, Birgit Steckel-Hamann, David Dexter, Nikul Bakshi, Joe Mather, Seleen Ong, Carla Cox, Yiorgos Christakis, Hao Zhang, Carrie Nothcott, Elaine Borthwick, David Nobbs, Jens Schjodt-Eriksen, Sebastian Holst, Florian Lipsmeier, Jason Hannon, Nadir Ammour, Haneen Njoum, Hillol Sarker, Imane Brigui, Raolat Abdulai, Xavier Benain, Jimena Diaz DeLeon, Manon Cariou, Fabrice Bonche, Vincent Mittoux, Sheila Thomas, Caroline Zutterling, Juliette Muszka, Frederique Guilbert, Xavier Brusson, Gwenaelle Corre, Babak Boroojerdi, Coralie Domange, Phil Scordis, Kasper Claes, Valentina Ticcinelli, Chengliang Dai, Giovanni Campana, Sarah Bilali, Oliver Stumpp, Mireille Delval, David Marquet, Gwenaelle de Keyser, Claudia Mazza, Alexandra Auffret, Jeremy Edgerton, Juha Rouru,Minna Korolainen, Sammeli Liikkanen, Mikko Kuoppamäki, Marina Lindford, Anssi Mäkiniemi, Toni Sarapohja, Olavi Kilkku, Antonella Chiucchiuini, Brian Tracey, Dimitri Volfson, Tairmae Kangarloo, Francesco Onorati, Wojtek Piwko, Geert van Gassen, Todd Swick, Armella Escher, Pietro Artoni, Robert Latzman, Mike Chambers, Elizabeth Amstutz, Nick Bott, Laura Rosen, Ieuan Clay, Aiden Doherty, Sara Riggare, Dina de Sousa, Cate Titterton, Heather Hunter, Ulli Funken, Jill Shutt, Werner Rammele, Jean Heather, Paul Howard.

## Author contributions

A-KB and DA conceived the idea. A-KB wrote the initial draft. All authors contributed to the article and revised it critically for important intellectual content and approved the submitted version.

## Funding

The **RADAR-AD** project has received funding from the Innovative Medicines Initiative 2 Joint Undertaking under grant agreement No 806999. This Joint Undertaking receives support from the European Union’s Horizon 2020 research and innovation programme and EFPIA and Software AG. See www.imi.europa.eu for more details. This communication reflects the views of the RADAR-AD consortium and neither IMI nor the European Union and EFPIA are liable for any use that may be made of the information contained herein. Research of Alzheimer Center Amsterdam is part of the neurodegeneration research program of Amsterdam Neuroscience. Alzheimer Center Amsterdam is supported by Stichting Alzheimer Nederland and Stichting Steun Alzheimercentrum Amsterdam. The **MOBILISE-D** project has received funding from the Innovative Medicines Initiative 2 Joint Undertaking under grant agreement No. 820820. This Joint Undertaking receives support from the European Union’s Horizon 2020 research and innovation programme and the European Federation of Pharmaceutical Industries and Associations (EFPIA). The **IDEA-FAST** project has received funding from the Innovative Medicines Initiative 2 Joint Undertaking under grant agreement No. 853981. This Joint Undertaking receives support from the European Union’s Horizon 2020 research and innovation programme and EFPIA and associated partners.

## Conflict of interest

AP received grant support from Ministry of Health (MINSAL) and Ministry of Education, Research and University (MIUR), from Airalzh Foundation, LIMPE-DSIMOV society and MI H2020 initiative (MI2-2018-15-06); he received speaker honoraria from Abbvie, Bial, Biomarin, Roche and Zambon Pharmaceuticals. W-FN has consulted for Novartis, GlaxoSmithKline, Abbvie, BMS, Sanofi, MedImmune, Janssen, Resolve Therapeutics and UCB. LR receives consultancy from MJ Fox Foundation and grant support from the EU, NIHR, MRC, PDUK, Dunhill Medical Trust, Cure Parkinson’s Trust, EPSRC, MJ Fox Foundation. DA has received research support and/or honoraria from Astra-Zeneca, H. Lundbeck, Novartis Pharmaceuticals, Evonik, Roche Diagnostics, and GE Health, and served as paid consultant for H. Lundbeck, Eisai, Heptares, Mentis Cura, Eli Lilly, Cognetivity, Enterin, Acadia, EIP Pharma, and Biogen. JC was employed by Novartis Institutes for Biomedical Research (NIBR), Basel, Switzerland and GE was employed by Novartis Pharmaceuticals Corporations, Cambridge, MA, United States.

The remaining authors declare that the research was conducted in the absence of any commercial or financial relationships that could be construed as a potential conflict of interest.

## Publisher’s note

All claims expressed in this article are solely those of the authors and do not necessarily represent those of their affiliated organizations, or those of the publisher, the editors and the reviewers. Any product that may be evaluated in this article, or claim that may be made by its manufacturer, is not guaranteed or endorsed by the publisher.

## Footnotes

^1^Muurling M, de Boer C, Hinds C, Atreya A, Doherty A, Alepopoulos V, et al. and the RADAR-AD Consortium. Feasibility and user experience of remote monitoring in Alzheimer’s disease. (submitted)

^2^ www.go-fair.org
